# Inhibitory Activity of Bevacizumab to Differentiation of Retinoblastoma Cells

**DOI:** 10.1371/journal.pone.0033456

**Published:** 2012-03-22

**Authors:** Jang Won Heo, Jin Hyoung Kim, Chang Sik Cho, Hyoung Oh Jun, Dong Hun Kim, Young Suk Yu, Jeong Hun Kim

**Affiliations:** 1 Department of Ophthalmology, Seoul National University College of Medicine, Seoul National University, Seoul, Korea; 2 Fight against Angiogenesis-Related Blindness (FARB) Laboratory, Clinical Research Institute, Seoul National University Hospital, Seoul, Korea; 3 Department of Radiology, College of Medicine, Sooncheonhyang University, Bucheon, Korea; 4 Tumor Microenvironment Research Center, Global Core Research Center, Seoul National University, Seoul, Korea; Yale Medical School, United States of America

## Abstract

Vascular endothelial growth factor (VEGF) is a major regulator in retinal and choroidal angiogenesis, which are common causes of blindness in all age groups. Recently anti-VEGF treatment using anti-VEGF antibody has revolutionarily improved the visual outcome in patients with vaso-proliferative retinopathies. Herein, we demonstrated that bevacizumab as an anti-VEGF antibody could inhibit differentiation of retinoblastoma cells without affection to cellular viability, which would be mediated *via* blockade of extracellular signal-regulated kinase (ERK) 1/2 activation. The retinoblastoma cells expressed VEGFR-2 as well as TrkA which is a neurotrophin receptor associated with differentiation of retinoblastoma cells. TrkA in retinoblastoma cells was activated with VEGF treatment. Interestingly even in the concentration of no cellular death, bevascizumab significantly attenuated the neurite formation of differentiated retinoblastoma cells, which was accompanied by inhibition of neurofilament and shank2 expression. Furthermore, bevacizumab inhibited differentiation of retinoblastoma cells by blockade of ERK 1/2 activation. Therefore, based on that the differentiated retinoblastoma cells are mostly photoreceptors, our results suggest that anti-VEGF therapies would affect to the maintenance or function of photoreceptors in mature retina.

## Introduction

As originally discovered as an endothelial-specific growth factor [1], vascular endothelial growth factor (VEGF) plays a critical role in physiological and pathological angiogenesis [2]. Interestingly, VEGF generated from the nervous system has been documented to function in neurons as well as endothelial cells [3]. VEGF seems to share common molecular triggers and signaling pathways in neurons and endothelial cells of the nervous system as our previous suggestion [4], [5]. Actually, the neurotrophic and neuroprotective effect of VEGF could be mediated through its tyrosine kinase receptor, VEGFR-2 which is widely expressed in neurons and glias [6]. With recent observations to unravel the direct effect of VEGF on neurons and glias, VEGF appears to be essential for neuronal development and survival in physiological and pathological conditions [7].

Retinal and choroidal neovascularization are common causes of blindness in all age groups - retinopathy of prematurity (ROP) is for children, diabetic retinopathy (DR) for young adults and age-related macular degeneration (AMD) for elderly [8]. Although other angiogenic factors could contribute to pathological angiogenesis in the eye, VEGF is a major regulator in retinal and choroidal angiogenesis [9]. With recent development of anti-VEGF therapy using anti-VEGF antibody, the visual outcome in patients with retinal and choroidal vascular diseases has been revolutionarily improved [10].

Bevacizumab is a full-length humanized monoclonal antibody to bind to all isoforms of VEGF-A, which was approved by the US Food and Drug Administration for metastatic colorectal cancer in combination with an intravenous 5-fluorouracil based regimen [11]. Without an approval for intraocular use of bevacizumab, its application has been progressively expanded based on empirical evidence in clinics [12], [13]. Now, bevacizumab is a promising therapeutic option targeting to VEGF-mediated vasoproliferative diseases in the retina [14]. However, based on the neurotrophic and neuroprotective effect of VEGF, concerns have been raised about neuronal toxicity in the retina following intravitreal injection of bevacizumab. Although consecutive reports showed little toxic effect of intravitral bevacizumab on the retina including ganglion cell, retinal neuron, and retinal pigment epithelial cell [14]–[17], a few reports demonstrated that intravitreal bevacizumab could induce retinal toxicity [18], [19]. Therefore, regardless of widespread use of bevacizumab, some issues for its biocompatibility and safety remains to be addressed.

In current study, we demonstrated that bevacizumab could inhibit differentiation of retinoblastoma cells under the concentration never affecting to cellular viability. The retinoblastoma cells expressed VEGFR-2 as well as TrkA which is a neurotrophin receptor associated with differentiation of retinoblastoma cells [20]. TrkA in retinoblastoma cells was activated with VEGF treatment. Interestingly even in the concentration of no cellular death, bevascizumab significantly attenuated the neurite formation of differentiated retinoblastoma cells, which was accompanied by inhibition of neurofilament and shank2 expression [21]. In addition, we showed that bevacizumab inhibits the differentiation of retinoblastoma cells by blockade of extracellular signal-regulated kinase (ERK) 1/2 activation. Taken together, it should be carefully concerned that bevacizumab treatment could attenuate differentiation of retinal neurons though not induces cell death.

## Materials and Methods

### Cell Culture

Human retinoblastoma cell lines, Y79 and SNUOT-Rb1, established by our group [22], and a human colorectal cancer cell line, SW480, were maintained in RPMI 1640 medium (Welgene Inc., Seoul, Korea) supplemented with 10% fetal bovine serum (Gibco BRL, Rockville, MD, USA) and 1% antibiotic-antimycotic solution (Invitrogen, Carlsbad, CA, USA) at 37°C in a moist atmosphere of 95% air and 5% CO2. The medium was changed every third day. Cultured tumor cells were observed daily under a phase-contrast microscope (Carl Zeiss, Chester, VA, USA). If needed, VEGF (10 ng/ml, Sigma, St. Louis, MO, USA) treatment was carried out. To induce the differentiation of retinoblastoma cells, 0.1% bovine serum albumin (BSA, Sigma-Aldrich, St Louis, MO, USA) supplied into the culture media up to 48 hours.

### Cell Viability Assay

Cell viability was determined by using a 3-(4, 5-dimethylthiazol-2-yl)-2, 5-diphenyltetrazolium bromide (MTT) assay. As our recent report [17], SNUOT-Rb1 (1×10^4^ cells) was plated in 96-well culture plates, and then treated with 0.1 to 10 mg/ml bevacizumab for 48 hours. The medium was then replaced with fresh medium containing 0.5 mg/ml MTT for 4 h. After incubation, the medium was carefully removed from the plate, and dimethyl sulfoxide was added to solubilize formazan produced from MTT by the viable cells. Absorbance was measured at 540 nm using a microplate reader (Molecular Devices, Sunnyvale, CA, USA).

### Measurement of Neurite Length in Differentiated Retinoblastoma Cells

As our recent report [23], neurite length in differentiated retinoblastoma cells was measured by manual tracing of neurite outgrowths in each cell. 15 to 20 retinoblastoma cells were evaluated in randomly selected fields at a magnification of ×400 and photographed with a digital camera (DXC 930 P, Sony, Tokyo, Japan) under an inverted microscope (Axiovert 200M, Carl Zeiss). For analysis, differentiated retinoblastoma cells were selected randomly and neurites were traced manually. Neurite length was measured from an arbitrary round line connecting edges of non-spiny cell membrane to the distal tip of the neurite. Every neurite was traced by a series of straight lines; each neurite can be mathematically described as a series of straight lines leading from the edge to the neurite tip. The length of each of these lines was determined by the curvatures of the neurite such that the line always overlaid the neurite. Accumulation of the straight line segments results in a polygon equivalent to the neurite length.

### Western Blot Analysis

Cells were harvested, washed with ice-cold phosphate buffer solution, and lysed with buffer containing 50 mM of Tris–HCl (pH 7.4), 150 mM of NaCl, 1% Nonidet P40, 2 mM of sodium orthovanadate, and a protease inhibitor cocktail (Roche). An equal amount (15 µg) of the samples was separated on sodium dodecylsulfate-polyacrylamide gel and then transferred onto nitrocellulose filters (Bio-Rad Laboratories, Hercules, CA, USA). The membranes were immunoblotted with primary antibodies against VEGFR-2 (1∶1000, Santa-Cruz Biotechnology, Santa Cruz, CA, USA), phospho-TrkA (1∶1000, Santa-Cruz Biotechnology, Santa-Cruz, CA, USA), TrkA (1∶1000, Santa-Cruz Biotechnology, Santa-Cruz, CA, USA), neurofilament (1∶1000, Chemicon, Temecula, CA, USA), shank 2 (1∶1000, Santa-Cruz Biotechnology, Santa Cruz, CA, USA), phospho-ERK 1/2 (1∶1000, Cell Signaling Technology, Beverly, MA, USA), ERK 1/2 (1∶1000, Cell Signaling Technology), phospho-Akt (1∶1000, Cell Signaling Technology), and Akt (1∶1000, Cell Signaling Technology). To ensure the equal loading of protein in each lane, the blots were stripped and reprobed with an antibody against β-actin.

### Immunocytochemistry

SNUOT-Rb1 was grown and seeded on Deckglaser coverslips (Carolina Biological, Burlington, NC, USA). Cells were fixed in 4% paraformaldehyde for over-night at 4°C. The primary antibodies against neurofilament (1∶100, Chemicon, Temecula, CA, USA) and shank2 (1∶100, Santa-Cruz Biotechnology, Santa Cruz, CA, USA) were diluted in PBS and added to the specimen followed by incubation for over-night at room temperature. Alexa Fluor 546 donkey anti-goat IgG (1∶400, Molecular probes, Eugene, OR, USA), Alexa Fluor 488 donkey anti-rabbit IgG (1∶400, Molecular probes, Eugene, OR, USA) were used as secondary antibodies. The nuclei were stained with 4′, 6-diamidino-2-phenolindole (DAPI, Sigma-Aldrich Co., St. Louis, MO, USA). The slides were mounted with Faramount Aqueous mounting medium (DAKO, Glostrup, Denmark) and observed under fluorescence microscope (Axio observer, Carl Zeiss, Chester, VA, USA).

### Reverse transcriptase-polymerase chain reaction (RT-PCR) analysis

Total RNA from cells was isolated using TRIzol reagent (Invitrogen, Carlsbad, CA, USA) according to the manufacturer's instructions. First-stranded cDNA was synthesized with 3 µg each of DNA-free total RNA and oligo-(dT) 16 primer by Moloney murine leukemia virus reverse transcriptase (Promega, Madison, WI, USA). Equal amounts of cDNA were subsequently amplified by PCR in a 50-µL reaction volume containing 1× PCR buffer; 200 µM of dNTPs; 10 µM of specific primer for *neurofilament* (5′-AAGCATAACCAGTGGCTACTCCCA-3′ and 5′-TCCTTGGCAGCTTCTTCCTCTTCA-3′), *shank2* (5′-GCGTGCATCCAAGAAATGCG-3′ and 5′-AGGTTCAGTAGACTCGAATGG-3′), and *GAPDH* (5′-TCCCTCAAGATTGTCAGCAA-3′ and 5′-AGATCCACAACGGATACATT-3′) and 1.25 U of Taq DNA polymerase (TaKaRa, Tokyo, Japan). Amplification was performed for a total of 25 to 35 cycles. To ensure the equal loading of mRNA in each lane, *GAPDH* expression was measured.

### Statistical Analysis

Statistical differences between groups were evaluated with the Student's unpaired t-test (two-tailed). Data were recorded as mean ± SD. P values≤0.05 were considered significant.

## Results

### Activation of TrkA Induced by VEGF in Retinoblastoma Cells

**Figure 1:**
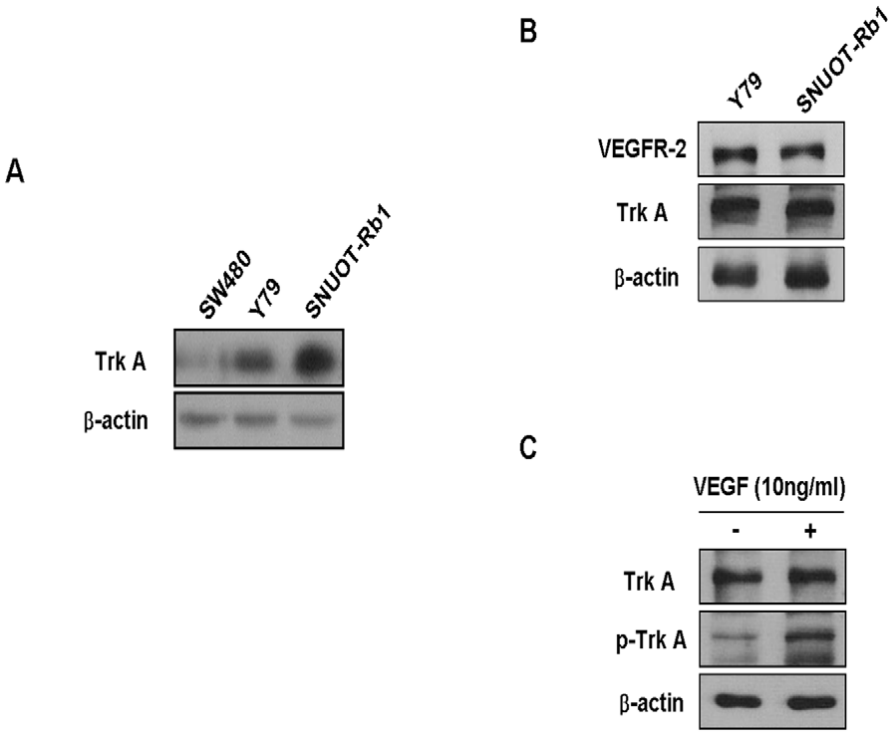
Activation of TrkA Induced by VEGF in Retinoblastoma Cells. (A) Proteins of human retinoblastoma cell lines, Y79 and SNUOT-Rb1 [22] as well as a human colorectal cancer cell lines, SW480 were resolved on 12% SDS-PAGE and western blot analysis was performed using anti-TrkA antibody. β-actin was served as a loading control. Each figure is representative ones from three independent experiments. (B) Proteins of Y79 and SNUOT-Rb1 cells were resolved on 12% SDS-PAGE and western blot analysis was performed using anti-VEGFR-2 and anti-TrkA antibody. β-actin was served as a loading control. Each figure is representative ones from three independent experiments. (C) SNUOT-Rb1 cells were treated with 10 ng/ml VEGF. TrkA and phospho-TrkA were detected by Western blot analysis. β-actin was served as a loading control. Each figure is representative ones from three independent experiments.

First, we examined whether TrkA is expressed in retinoblastoma cells. Compared to a colorectal cancer cell line, SW480 without Trk A expression as a negative control [24], TrkA was highly expressed in both retinoblastoma cell lines of Y79 and SNUOT-Rb1. (Figure 1A) Then, to determine whether VEGFR-2 and TrKA, as a neurotrophin receptor are expressed in retinoblastoma cells, the expression of VEGFR-2 and TrkB was measured in retinoblastoma cell lines, Y79 and SNUOT-Rb1, by western blot analysis. As demonstrated in figure 1B, TrkA as well as VEGFR-2 were expressed in high level in both retinoblastoma cell lines. As depicted in our recent report [20], neurotrophin receptors including TrkA and TrkB are differentially expressed in retinoblastoma cells depending on cellular differentiation status. In particular, TrkA expression is related to differentiation of retinoblastoma cells. Therefore, it is interesting to note that the addition of exogenous VEGF effectively induced phosphorylation of TrkA in retinoblastoma cells. (Figure 1C).

### Attenuation of Neurite Outgrowth by Bevacizumab in Differentiated Retinoblastoma Cells

**Figure 2:**
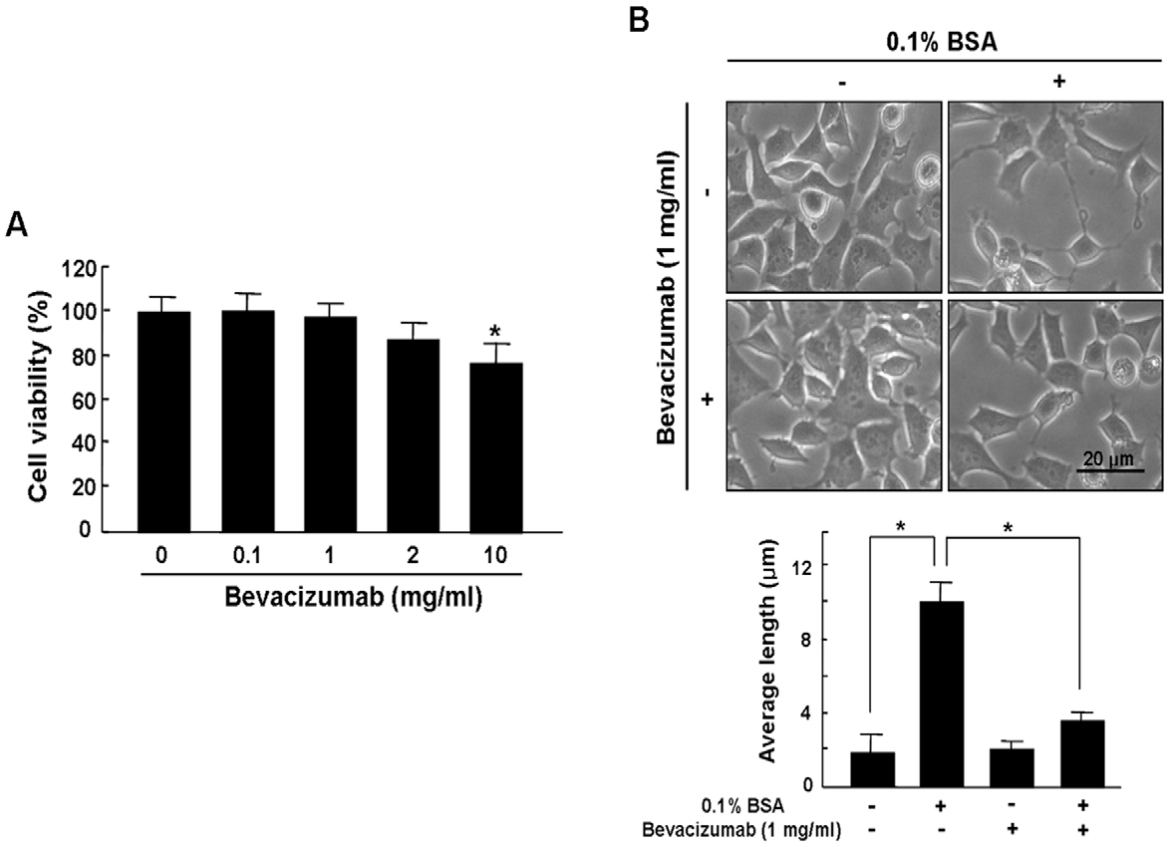
Attenuation of Neurite Outgrowth by Bevacizumab in Differentiated Retinoblastoma Cells. (A) SNUOT-Rb1 cells were treated with 0.1 to 10 mg/ml bevacizumab. Cell viability was measured by MTT [3-(4, 5-dimethylthiazol-2-yl)-2, 5-diphenyltetrazolium bromide] assay. Quantitative analysis was performed by measuring viable cells relative to the controls. Each value represents means ± SE from three independent experiments (*P<0.05). (B) SNUOT-Rb1 cells were treated with either 0.1% BSA or 1 mg/ml bevacizumab. Neurite length in differentiated retinoblastoma cells was measured by manual tracing of neurite outgrowth in each cell. The figures that appear here were selected as representative of data from three independent experiments. Quantitative analysis was performed using average length from total neurites measured. Each value represents means ± SE from three independent experiments (*P<0.05). Scale bar, 20 µm. BSA, bovine serum albumin.

To investigate cytotoxic effect of bevacizumab on retinoblastoma cells, cell viability was evaluated through MTT assay in various concentrations of bevacizumab (0.1∼10 mg/ml). As demonstrated in figure 2A, the viability of retinoblastoma cells treated with bevacizumab was not affected up to 2 mg/ml as our recent report [17], whereas at a concentration of 10 mg/ml cellular viability of retinoblastoma cells was significantly decreased. (*P<0.05) Next, to investigate whether blockade of VEGF by bevacizumab could inhibit differentiation of retinoblastoma cells, we measured neurite length in differentiated retinoblastoma cells incubated with 0.1% BSA. 1 mg/ml bevacizumab was applied to exclude inhibition of neurite outgrowth from cytotoxicity findings for retinoblastoma cells. As shown in figure 2B, neurite outgrowth significantly increased with 0.1% BSA, which was, however, significantly attenuated by bevacizumab treatment.

### Inhibition of Neurofilament and Shank2 Expression by Bevacizumab in Differentiated Retinoblastoma Cells

**Figure 3:**
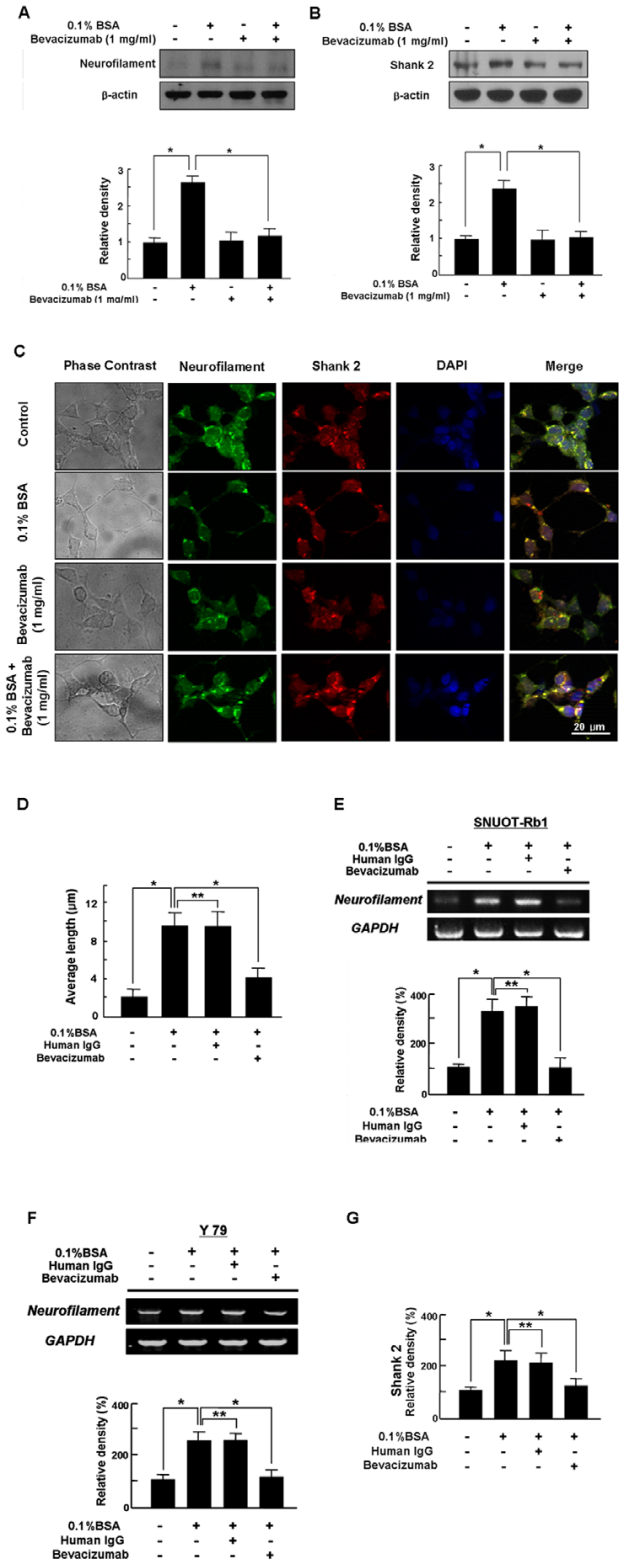
Inhibition of Neurofilament and Shank2 Expression by Bevacizumab in Differentiated Retinoblastoma Cells. (A, B) SNUOT-Rb1 cells were treated with 0.1% BSA or 1 mg/ml bevacizumab. Neurofilament (A) and shank2 (B) were detected by Western blot analysis. β-actin was served as a loading control. Each figure is representative ones from three independent experiments. Quantitative analysis was performed by measuring protein expression relative to the controls. Each value represents means ± SE from three independent experiments (*P<0.05). BSA, bovine serum albumin. (C) SNUOT-Rb1 cells were treated with 0.1% BSA or 1 mg/ml bevacizumab. Neuronal differentiation was addressed by the morphological changes of neurite extensions. Immunocytochemistry for neurofilament (green) and shank2 (red) was performed, and nuclei were labeled with DAPI (blue). Each figure is representative ones from three independent experiments. Scale bar, 20 µm. DAPI, 4′, 6-diamidino-2-phenolindole. (D) SNUOT-Rb1 cells were treated with 0.1% BSA, 1 mg/ml human IgG, or 1 mg/ml bevacizumab. Neurite length in differentiated retinoblastoma cells was measured by manual tracing of neurite outgrowth in each cell. Quantitative analysis was performed using average length from total neurites measured. Each value represents means ± SE from three independent experiments (*P<0.05, **P>0.05). (E, F, G) Retinoblastoma cells of SNUOT-Rb1 (E) and Y79 (F, G) were treated with either 0.1% BSA or 1 mg/ml human IgG. Total mRNA was isolated from retinoblastoma cells, and reverse transcriptase-polymerase chain reaction was performed with specific primers for neurofilament or shank2. GAPDH was served as an internal control. Each figure is representative ones from three independent experiments. Quantitative analysis was performed by measuring mRNA expression relative to the control. Each value represents means ± SE from three independent experiments (*P<0.05, **P>0.05).

Given that shank 2 is expressed in the retina and colocalized with neurofilament of a neuronal differentiation marker, which is confirmed by its colocalization with neurofilament at the dendritic region of the differentiated retinoblastoma cells [21], neurofilament and shank2 expression were investigated to confirm whether bevacizumab inhibits differentiation of retinoblastoma cells. We used Western blotting to measure expression of neurofilament and shank2 in differentiated retinoblastoma cells induced by 0.1% BSA. As shown in figure 3A, up-regulation of neurofilament in differentiated retinoblastoma cells was significantly inhibited by 1 mg/ml bevacizumab. Expectedly, shank2 expression was significantly increased with treatment of 0.1% BSA, which was nearly suppressed by bevacizumab treatment. (Figure 3B)

To confirm whether bevacizumab-induced shrinkage of neurite outgrowth is related to loss of neurofilament and shank2 expression on outgrowing neurites, we performed immunocytochemistry for neurofilament and shank2 in differentiated retinoblastoma cells. As our recent report [21], neurofilament as well as shank2 were diffusely expressed around the nucleus in undifferentiated retinoblastoma cells, whereas they were prominently expressed at the contacts of outgrowing neurites in differentiated cells. (Figure 3C) However, neurite outgrowth was attenuated by bevacizumab treatment, which was accompanied by diffuse expression of neurofilament and shank2 around the nucleus as undifferentiated cells. (Figure 3C).

Next, Y79 cell line, another retinoblastoma cell line was used to ensure that our provided results are not SNUOT-Rb1 specific responses. In addition, human IgG was used as a non-specific antibody to show that the response is not induced by non-specific antibody. As shown in figure 3D, we measured neurite length in differentiated retinoblastoma cells incubated with 0.1% BSA. Neurite outgrowth significantly increased with 0.1% BSA, which was, however, significantly attenuated by bevacizumab treatment. (* P<0.05) However, human IgG treatment never affected to neurite outgrowth induced by 0.1% BSA. (** P>0.05) In addition, neurofilament expression in SNUOT-Rb 1 cells was significantly increased with treatment of 0.1% BSA to induce the differentiation of retinoblastoma cells, (* P<0.05) which was not affected by human IgG treatment. (** P>0.05) However, the up-regulation of neurofilament was completely inhibited by bevacizumab. (* P<0.05) (Figure 3E).

Similar to SNUOT-Rb1, 0.1% BSA-induced expression of neurofilament in Y79 retinoblastoma cells (* P<0.05) was not suppressed by human IgG treatment, (** P>0.05) which was significantly inhibited by bevacizumab. (* P<0.05) (Figure 3F) Furthermore, shank2 expression in Y79 cells was increased with treatment of 0.1% BSA, (* P<0.05) which was not affected by human IgG treatment. (** P>0.05) However, its up-regulation was effectively inhibited by bevacizumab. (* P<0.05) (Figure 3G).

### Bevacizumab-induced Inhibition of Differentiation of Retinoblastoma Cells through Blockade of ERK 1/2 Activation

**Figure 4:**
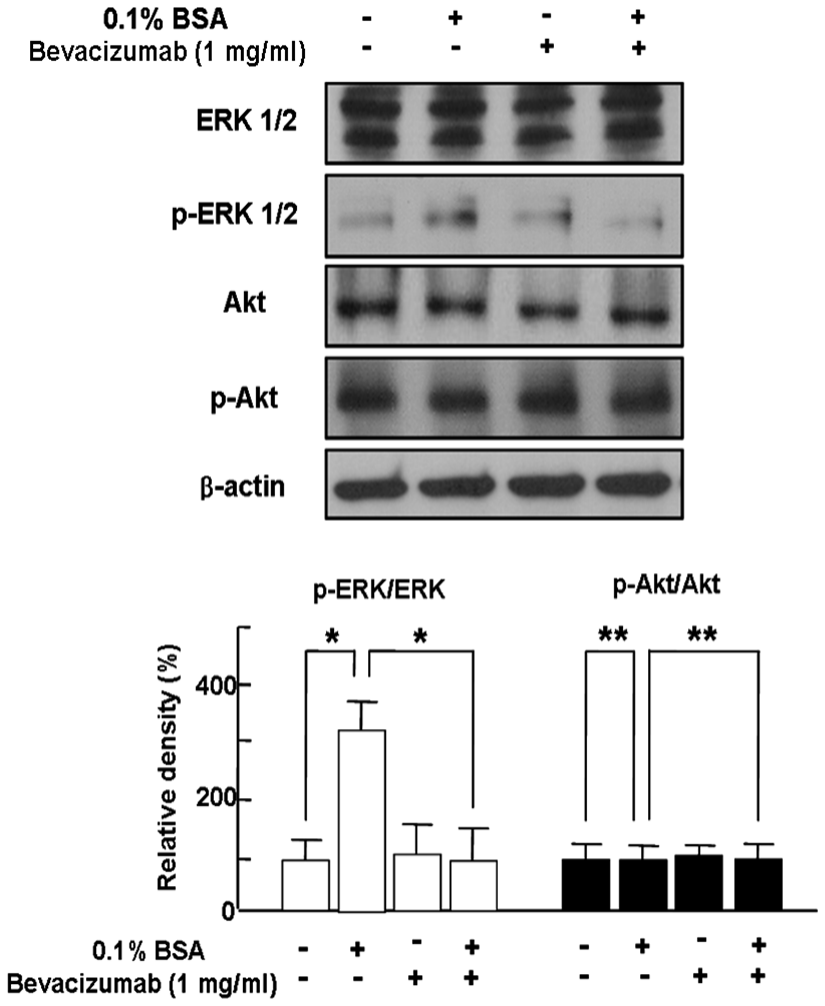
Bevacizumab-induced Inhibition of Differentiation of Retinoblastoma Cells through Blockade of ERK 1/2 Activation. SNUOT-Rb1 cells were treated with 0.1% BSA or 1 mg/ml bevacizumab. ERK 1/2, phospho-ERK 1/2, Akt, and phospho-Akt were detected by Western blot analysis. β-actin was served as a loading control. Each figure is representative ones from three independent experiments. Quantitative analysis was performed by measuring protein expression relative to the controls. Each value represents means ± SE from three independent experiments (*P<0.05, **P>0.05). BSA, bovine serum albumin.

Base on our reports that differentiation of retinoblastoma cells would be mediated by ERK 1/2 activation [23], [25], we addressed whether ERK 1/2 activation is changed in differentiated retinoblastoma cells by 1 mg/ml bevacizumab. As demonstrated in figure 4, phospho-ERK 1/2 was significantly increased in differentiated retinoblastoma cells, which was completely inhibited by bevacizumab treatment. However, there was no change in expression of Akt and phospho-Akt in differentiated retinoblastoma cell with bevacizumab treatment. (Figure 4).

## Discussion

Herein, we clearly demonstrated that bevacizumab treatment could inhibit differentiation of retinoblastoma cells without any change in cellular viability, which would be mediated via blockade of ERK 1/2 activation.

VEGF is considered as the most critical regulator of angiogenesis in physiological as well as pathological conditions, which is evidently mediated by the high-affinity cell surface receptors VEGFR-1 and VEGFR-2 [1], [2]. Although VEGF could bind to its receptors with high affinity, essential angiogenic processes are activated by VEGFR-2 transmission whereas VEGFR-1 indirectly modulates VEGFR-2 responses by decoying VEGF [26]. Accordingly, disturbance in VEGF-VEGFR-2 pathway causes severe vascular defects, which could be therapeutically applied to tumor angiogenesis and vaso-proliferative retinopathies [2], [25]. Therefore, regardless of empirical evidence in clinics, anti-VEGF treatment using bevacizumab has been extensively applied in variable vaso-proliferative retinopathies [12]–[14]. Some clinical reports have demonstrated that intravitreal bevacizumab would be most effective against angiogenic and edematous ocular diseases [27], [28]. However, because VEGF could play as a neurotrophic and neuroprotective factor in nervous system as well as critical roles in vessels, VEGF inhibition could lead to serious neuroretinal damages [3]–[7]. In the retina, VEGF and VEGFR-2 are expressed throughout most of neural retina including neurons, astrocytes, pericytes and Müller cells, which suggest that VEGF would be critical for more than its vascular roles [29]. As a result, systemic VEGF neutralization induces retinal degeneration through inhibition of VEGF-mediated neuroprotective effect on retinal neurons [29]. In contrast, a recent report provides that even long-term blockade of VEGF signaling could be applied in the retina without any toxicity to retinal cells [30]. Given opposite conclusions at controversy, it should be carefully investigated whether VEGF antagonism affect to retinal toxicity.

Interestingly, we found out that TrkA, a neurotrophin receptor, expressed on retinoblastoma cells can be phosphorylated by addition of VEGF, which is supported by a report that endothelial cell-derived VEGF enhances adult neurogenesis in a neurotrophin-dependent manner [31]. Considering our recent report that TrkA is involved in differentiation of retinoblastoma cells [20], This VEGF-induced activation of TrkA strongly encourage to investigate the effect of VEGF blockade on differentiation of retinoblastoma cells. Expectedly, VEGF neutralization using bevacizumab could lead to inhibition of differentiation of retinoblastoma cells without any affection to cellular viability. Bevacizumab treatment significantly attenuated neurite outgrowths in differentiated retinoblastoma cells. In neuronal differentiation, neurite outgrowth followed by axonal elongation and dendritic arborization is a fundamental morphological characteristic, which could be accompanied by neurofilament in axonal compartment and shank2, as a synaptic protein in dendritic area [20]. With treatment of bevacizumab, attenuation of neurite outgrowth in differentiated retinoblastoma cells was followed by inhibition of up-regulation of neurofilament and shank expression.

Although VEGF and nerve growth factor (NGF) as a neurotrophin were originally discovered to be specific for endothelial and neuronal cells, respectively, recent insights indicate that VEGF and NGF directly exert cross talk between nervous and vascular system [3], [4]. In detail, stimulation by VEGF and NGF can activate common intracellular signaling pathways of phosphoinositide 3-kinase (PI3K)/Akt and mitogen-activated protein kinase (MAPK)/ERK pathway [6], [7]. We clearly showed that VEGF neutralization was significantly inhibited differentiation of retinoblastoma cells via blockade of ERK pathway, which is strongly supported by our recent reports [20], [23], [25].

In conclusion, our data suggests that VEGF inhibition could affect to differentiation of retinoblastoma cells though no cellular toxicity. Based on that the differentiated retinoblastoma cells are mostly photoreceptors [32], our result provides that anti-VEGF therapies would affect to the maintenance or function of photoreceptors in mature retina, which is supported by a recent report that the neutralization of endogenous VEGF leads to unexpected neural toxicity [29]. Therefore, regardless of recent reports to demonstrate few clinically relevant ocular side effect [27], [28], pros and cons of anti-VEGF treatment should be carefully scrutinized.
