# Correction: Inhibitory Activity of Bevacizumab to Differentiation of Retinoblastoma Cells

**DOI:** 10.1371/annotation/008b05a8-229b-4aca-94ae-91e6dd5ca5ba

**Published:** 2012-05-15

**Authors:** Jang Won Heo, Jin Hyoung Kim, Chang Sik Cho, Hyoung Oh Jun, Dong Hun Kim, Young Suk Yu, Jeong Hun Kim

The fifth author's affiliations are incomplete. Dong Hun Kim is affiliated with both

3 Department of Radiology, College of Medicine, Sooncheonhyang University, Bucheon, Korea,

and

5 Department of Radiology, College of Medicine, Chosun University, Gwangju, Korea 

